# Arresting calcium-regulated sperm metabolic dynamics enables prolonged fertility in poultry liquid semen storage

**DOI:** 10.1038/s41598-023-48550-2

**Published:** 2023-12-08

**Authors:** Pangda Sopha Sushadi, Maiko Kuwabara, Ei Ei Win Maung, Mohamad Shuib Mohamad Mohtar, Kouyo Sakamoto, Vimal Selvaraj, Atsushi Asano

**Affiliations:** 1 Graduate School of Life and Environmental Sciences, University of Tsukuba, 1-1-1 Tennodai, Tsukuba, Ibaraki 305-8572 Japan; 2 Department of Animal Science, College of Agriculture and Life Sciences, Cornell University, Ithaca, NY 14853 USA; 3 Faculty of Life and Environmental Sciences, University of Tsukuba, 1-1-1 Tennodai, Tsukuba, Ibaraki 305-8572 Japan

**Keywords:** Reproductive biology, Energy metabolism, Animal physiology

## Abstract

The preservation of liquid semen is pivotal for both industrial livestock production and genetic management/conservation of species with sperm that are not highly cryo-tolerant. Nevertheless, with regard to poultry semen, even brief in vitro storage periods can lead to a notable decline in fertility, despite the in vivo capacity to maintain fertility for several weeks when within the hen’s sperm storage tubules. For fertility in sperm, intracellular calcium ions ([Ca^2+^]i) play a key role in signaling towards modifying energy metabolism. While reducing [Ca^2+^]i has been found to enhance the preservation of sperm fertility in some mammals, the connection between semen fertility and calcium availability in avian sperm has received limited attention. In this study, we demonstrate that the use of extracellular and intracellular calcium chelators in liquid semen extenders, specifically EGTA and EGTA-AM, has distinct effects on prolonging the fertility of chicken sperm. These results were validated through in vivo fertility tests. Mechanistically, the effects observed were linked to coordination of mitochondrial metabolism and ATP catabolism. Despite both calcium chelators inducing hypoxia, they differentially regulated mitochondrial respiration and ATP accumulation. This regulation was closely linked to a bimodal control of dynein ATPase activity; a direct initial activation with reduction in [Ca^2+^]i, and subsequent suppression by cytoplasmic acidification caused by lactic acid. These findings not only contribute to advancing poultry liquid semen preservation techniques, but also elucidates biologically relevant mechanisms that may underlie storage within the female reproductive tract in birds.

## Introduction

Effective semen preservation is crucial for promoting industrial livestock production and conserving their genetic resources. Semen storage at low temperatures slows metabolic rates and bacterial growth in semen diluents that mimic the composition of seminal plasma^1^, thereby prolonging viability and fertilization potential in vitro. Optimization of liquid semen storage conditions has been performed for various domestic animals, enabling semen fertility to be maintained for 3–10 days in boars, 1–3 days in bulls, and 1–2 days in stallions during in vitro storage^2^.

Liquid semen storage is particularly important in poultry sperm owing to its lower cryotolerance with freezing compared to mammalian sperm^1,3–5^. However, within 6–24 h a significant decline in semen fertility has been documented in chickens as well as turkeys^6,7^. In vivo within sperm storage tubules (SSTs) in proximity to the utero–vaginal junction (UVJ), it is well-known that ejaculated avian sperm can exist and be viable for extended periods with fertilization potential upto 3 weeks in chickens^8^ and 16 weeks in turkeys^9^. Numerous mechanistic studies on in vivo storage of avian sperm have suggested that optimizing semen storage conditions in poultry can be achieved by mimicking the extracellular or intracellular milieu of sperm residing in SSTs^10–13^. Similar to birds, in some mammals, a sperm reservoir in the lower oviduct could act as a sperm storage site that prolongs sperm viability and fertilizability. Investigating the mechanisms underlying storage-dependent fertility loss in boar sperm have uncovered associations with temperature, membrane destabilization, lipid peroxidation and calcium homeostasis disruption^14,15^. Particularly, reductions in intracellular calcium ([Ca^2+^]_i_) in mammalian sperm and as a result blocking premature capacitation-associated changes have prolonged fertility preservation^16^.

The regulation of [Ca^2+^]_i_ plays a crucial role in sperm motility and the acrosomal reaction (AR), which is essential for oocyte penetration. Using both genetic labeling and pharmacological methods, it was demonstrated that excessive [Ca^2+^]_i_ levels in mammalian sperm can be linked to impaired fertilization potential, including mitochondrial failure leading to a loss of motility^17^, activation of apoptotic cascades^18^, and premature loss of the acrosome, known as spontaneous AR (sAR)^19^. Sperm mitochondria play a vital role in coordinating ATP production, redox equilibrium, and calcium homeostasis, all of which can contribute to the regulation of sperm fertilization potential. Indicating a cross-genera conserved relevance, events that may be connected to mitochondrial impairments such as increased [Ca^2+^]_i_ levels, accumulation of reactive oxygen species (ROS), and decrease in mitochondrial membrane potential (ΔΨ_M_) have been demonstrated to occur in the early stages of both liquid storage in chicken^20,21^ and boar^22,23^ semen. Nevertheless, specific mechanistic associations between increased [Ca^2+^]_i_ levels and reduced semen quality during storage or impact on mitochondrial function remain unknown.

Poultry semen extenders have been developed without Ca^2+^ in their composition^6,24^. Despite this, and additional step of external calcium chelation using tetrasodium 1,2-bis-(*o*-aminophen-oxy)ethane-*N,N,N',N'-*tetraacetic acid (BAPTA) has been found to add to significantly sustaining chicken sperm motility during 5 h of storage at 10 °C^25^. However, this additional Ca^2+^ chelation did not result in full fertility sustenance in vivo when used for insemination with mobile sperm equivalents to fresh semen, indicating the existence of a possible multifactorial mechanism that drives functional impairment of chicken sperm during storage^25^. In stallion sperm, chelation of [Ca^2+^]_i_ using a membrane-permeable BAPTA analog (BAPTA-AM) prolonged progressive motility in stallion sperm for 48 h of semen storage at 5 °C without compromising in vivo fertility^26^. Although there could be species distinctions, experimental variations and differences to limiting extracellular vs intracellular Ca^2+^ availability, these studies support an engendering role for inhibiting calcium-driven events in semen fertility during storage. This premise indicates the need for controlled experiments to evaluate the precise outcomes and attempt to uncover how calcium chelation preserves sperm function.

The present study investigates the effect of arresting Ca^2+^- based signaling through chelation on multiple fertility parameters of chicken sperm during liquid storage, focusing largely on bioenergetic regulation and metabolism. Our findings demonstrate that both intracellular and extracellular Ca^2+^- specific chelation during liquid storage prolongs motility, promotes in vitro inner perivitelline layer (IPVL) penetration ability, and also sustained in vivo fertility. Metabolic state analyses revealed differences in the mechanisms underlying preservation of sperm fertilizability between intracellular and extracellular Ca^2+^ chelation, particularly in mitochondrial oxidative phosphorylation (OxPhos) and ATP catabolism regulation. These findings provide new insight into mechanisms that prolong poultry sperm fertilization potential during liquid storage, and benefit applications in genetic resource management for the poultry industry.

## Results

### Ca^2+^ chelation prolongs period of sperm viability and motility

**Figure 1:**
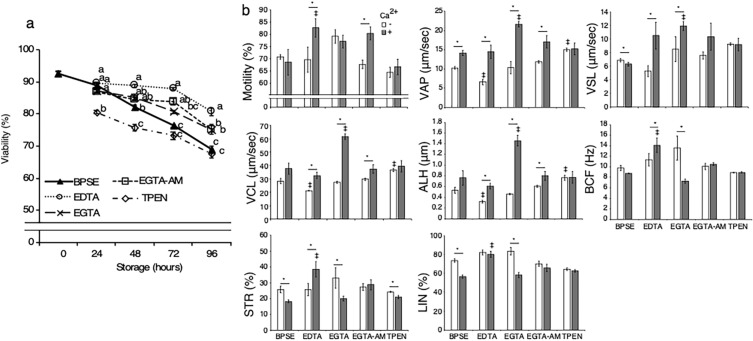
Chicken sperm viability and motility during liquid storage. Sperm were incubated at 4 °C for 0–96 h in BPSE supplemented with 2 mM EDTA, 2 mM EGTA, 10 µM EGTA-AM, or 10 µM TPEN, and subjected to viability assay (**a**). Viability decreased in a storage period–dependent manner, with a notable reduction under BPSE and TPEN conditions (^a–c^*P* < 0.05 in storage periods). Poststorage sperm of 72 h were analyzed using SMAS after activation with 5 mM Ca^2+^ addition (**b**). Multiple motility parameters in the EDTA, EGTA, and EGTA-AM groups were enhanced upon Ca^2+^ addition, showing significant differences compared with the control (BPSE) (^*^P < 0.05 in treatments; ^‡^*P* < 0.05 vs BPSE alone). Data are expressed as means ± SEM (n = 5, respectively).

The effect of different chelators on sperm viability in vitro was examined at 0–96 h poststorage. The viability of sperm at the start of incubation averaged 92.6%. With increasing storage time, viability declined in all treatments (Fig. 1a). At 24 h of storage, no significant difference was observed among the BPSE (control), EDTA, EGTA, and EGTA-AM groups. However, after 48 h, the EDTA, EGTA, and EGTA-AM groups showed significantly higher viability compared with the BPSE and TPEN groups. Viability for all timepoints and all treatment conditions remained > 68%, with the lowest being the BPSE and TPEN groups at 96 h.

In a previous report, another chelator BAPTA temporally and reversibly decreased sperm motility during storage, that could be recovered by Ca^2+^ addition after 5 h^25^. As we observed positive changes in our extended timepoints, we examined sperm motility profile at 72 h of storage after incubation with or without 5 mM Ca^2+^ for 10 min at 39 °C. Without Ca^2+^ addition, there were no significant differences in motility (64.4%–79.0%), VSL (5.3–9.2 μm/s), BCF (8.9–13.6 Hz), STR (24.2–33.0%), and LIN (64.5–83.5%) between the groups (Fig. 1b). However, higher values for VAP, VCL, and ALH were observed in the TPEN group, and a lower VCL (21.2) was observed in the EDTA group compared with the respective BPSE groups. The addition of Ca^2+^ increased motility parameters within the following groups: motility in EDTA (69.5 vs. 82.6%) and EGTA-AM (67.6 vs. 80.4%); VAP in BSPE (10.3 vs. 14.1 μm/s), EDTA (6.6 vs. 14.4 μm/s), EGTA (10.3 vs. 21.6 μm/s), and EGTA-AM (11.8 vs. 17.1 μm/s); VSL in EDTA (5.3 vs. 10.5 μm/s) and EGTA (8.5 vs. 11.9 μm/s); VCL in EDTA (21.2 vs. 32.5 μm/s), EGTA (27.3 vs. 61.7 μm/s), and EGTA-AM (29.8 vs. 37.3 μm/s); ALH in EDTA (0.3 vs. 0.6 μm), EGTA (0.5 vs. 1.6 μm), and EGTA-AM (0.6 vs. 0.8 μm); BCF in EDTA (11.3 vs. 14.1 Hz); and STR in EDTA (25.8 vs. 38.5%). These findings are consistent with reversible inactivation of sperm mobility with liquid storage for periods much longer than that previously reported for avian sperm^25^. Furthermore, the increases in motility in the EDTA group and for VAP, VSL, VCL, and ALH in the EGTA group were higher than the respective increases observed in the BPSE groups. In addition to 72 h storage, sperm motility profile was also examined at 24 and 48 h, which confirmed the significant reversible inactivation of sperm mobility consistently, albeit to a lesser extent appropriate for the shorter storage periods (Supplementary Fig. S1 and 2). Taken together, these results indicate that Ca^2+^ chelation potentiates the viability and motility preservation of chicken sperm during storage.

### Ca^2+^ chelation preserves sperm function and in vivo fertility

**Figure 2:**
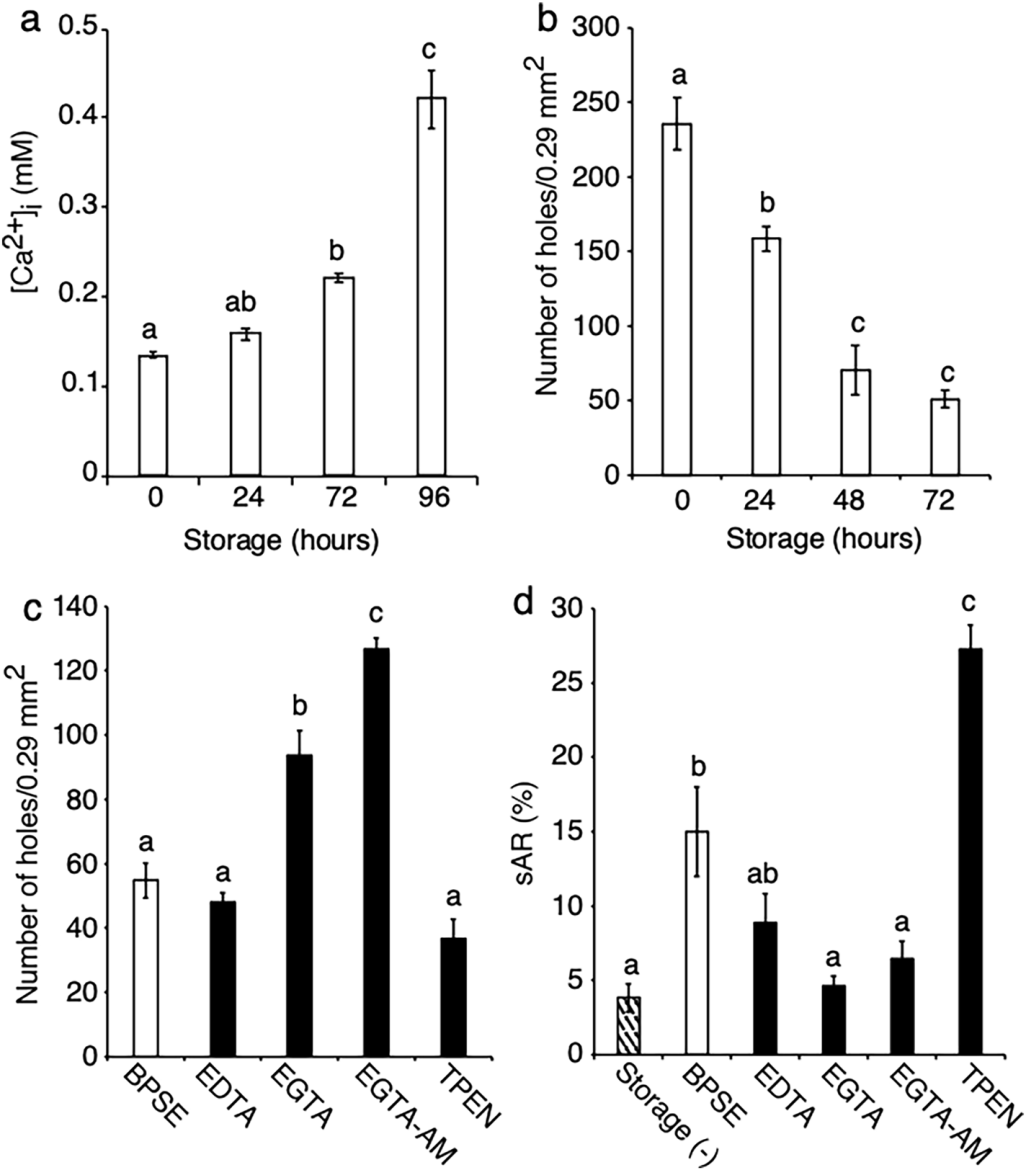
Changes in [Ca^2+^]i, IPVL penetrability, and spontaneous AR in chicken sperm during liquid storage. Sperm were incubated at 4 °C in BPSE for 0, 24, 72, or 96 h, followed by [Ca^2+^]i quantification and an IPVL penetration assay. [Ca^2+^]i levels increased and IPVL penetrability decreased in a storage period–dependent manner (**a** and **b**). At 72 h poststorage, IPVL penetrability and spontaneous AR were examined in groups treated with 2 mM EDTA, 2 mM EGTA, 10 µM EGTA-AM, or 10 µM TPEN. EGTA and EGTA-AM prolonged IPVL penetrability (**c**) while inhibiting spontaneous AR (**d**). Data are expressed as means ± SEM (n = 5, respectively). ^a–d^*P* < 0.05.

The effect of storage period on sperm fertilization potential was assessed by examining Ca^2+^ permeability and IPVL penetrability during the post storage period (timepoints across 0–96 h). Incubation of sperm in the presence of 1 mM Ca^2+^ for 15 min, followed by preloading of the fluorescent calcium indicator Fluo 3-AM, led to an increase in [Ca^2+^]i and a decrease in IPVL penetrability in a storage period–dependent manner (Fig. 2a,b). Elevated [Ca^2+^]i enables chicken sperm to undergo AR without physiological stimulation, i.e., spontaneous AR^27^. Therefore, we examined the effect of Ca^2+^ chelation on IPVL penetration and AR at 72 h storage. EGTA and EGTA-AM significantly prolonged penetrability (93.8 and 126.8 holes; *P* < 0.05) compared to BPSE (control), EDTA, and TPEN (54.8, 48.0, and 36.8 holes, respectively; Fig. 2c). Although EGTA and EGTA-AM decreased spontaneous AR to the same level as that of fresh sperm [storage (-)], EDTA did not have such an effect (Fig. 2d). Together with the results from viability and motility assays, these findings indicate that specific Ca^2+^ chelation promotes the preservation of IPVL penetrability.

In vivo fertility was examined at 48 h storage with EGTA or EGTA-AM using intravaginal artificial insemination. Findings from the trial showed no significant difference between treatments during the 1st week (Table 1). However, higher fertility was observed under Ca^2+^ chelation compared to the control during the 2nd week (*P* < 0.017), with a particularly potent effect observed in the EGTA group. The EDTA group was not used for testing in vivo fertility as there was no increase in IPVL sperm penetration as noted for the EGTA and EGTA-AM groups. These results indicate that intracellular and extracellular Ca^2+^ chelation using EGTA and EGTA-AM can preserve chicken sperm functions and in vivo fertilizability during storage.

**Table 1:** Effect of calcium chelators on in vivo sperm fertility at 48 h of the post-storage.

Addition	Post-AI	% of fertility (no. of fertile/incubated eggs)					
		Trial 1	Trial 2	Trial 3	Trial 4^#^	Trial 5^#^	Total
Control	1st wk	100.0 (21/21)	100.0 (21/21)	100.0 (21/21)	88.2 (15/17)	77.8 (14/18)	93.2 ± 3.7
EGTA	1st wk	85.7 (18/21)	100.0 (21/21)	100.0 (21/21)	94.7 (18/19)	95.2 (20/21)	95.1 ± 2.1
EGTA-AM	1st wk	85.7 (18/21)	100.0 (21/21)	100.0 (21/21)	92.6 (25/27)	88.5 (23/26)	93.4 ± 2.4
Control	2nd wk	38.1 (8/21)	19.0 (4/21)	4.8 (1/21)	25.0 (5/20)	33.3 (6/18)	24.0 ± 4.8^a^
EGTA	2nd wk	57.1 (12/21)	81.0 (17/21)	71.4 (16/21)	65.0 (13/20)	57.9 (11/19)	66.5 ± 3.6^b^
EGTA-AM	2nd wk	57.1 (12/21)	61.9 (13/21)	42.9 (9/21)	46.4 (13/28)	42.3 (11/26)	50.1 ± 3.2^c^

Data are presented as mean ± SEM (*n* = 5). ^#^4 hens were used for EGTA-AM treatment group. ^a–c^*P* < 0.05 in same post-AI wk.

### Differential regulation of mitochondrial oxidative phosphorylation by extracellular and intracellular Ca^2+^ chelation

**Figure 3:**
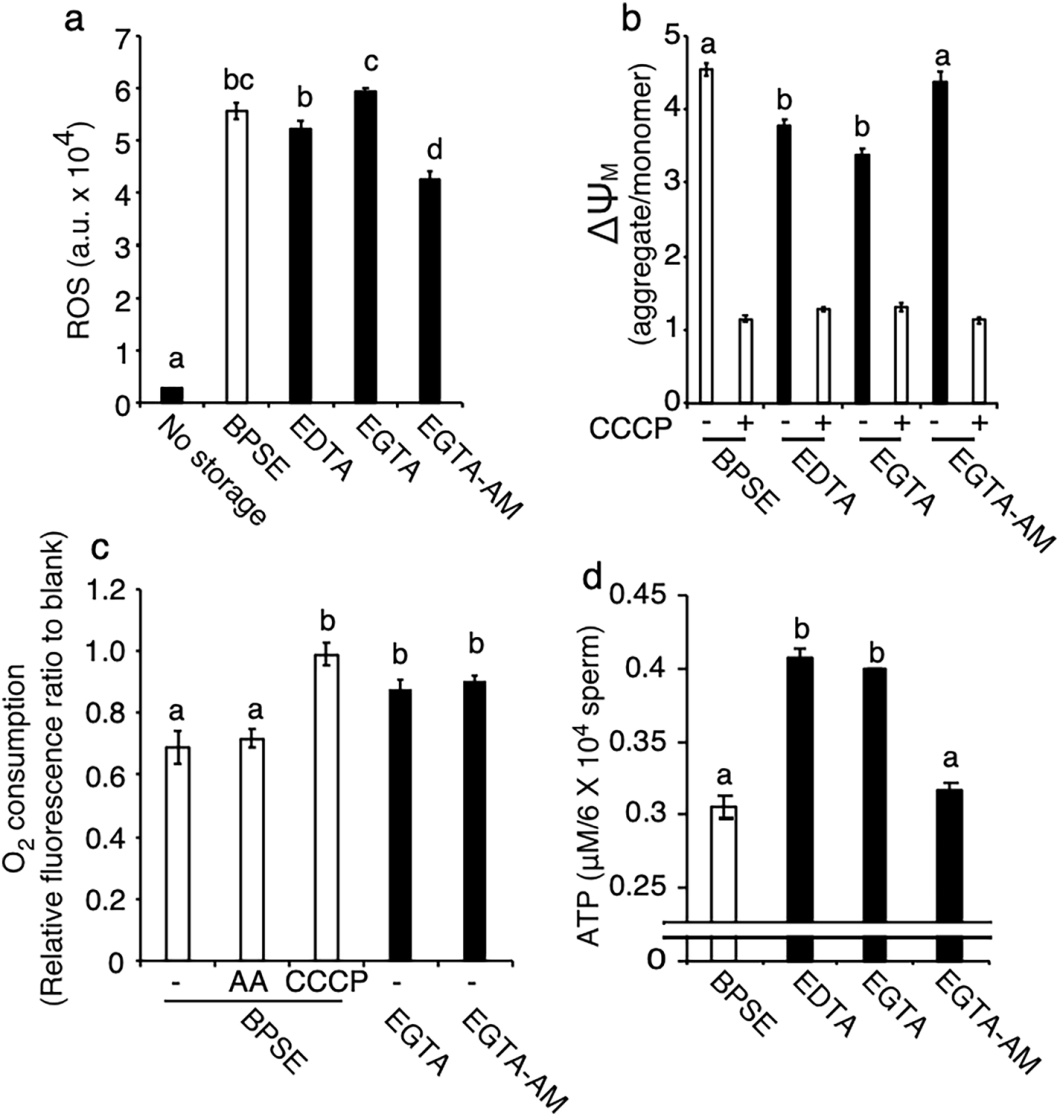
Mitochondrial redox and OxPhos of chicken sperm during liquid storage. Sperm were incubated at 4 °C for 72 h with 2 mM EDTA, 2 mM EGTA, or 10 µM EGTA-AM and subjected to quantification of ROS, ΔΨ_M_, oxygen consumption and ATP levels. ROS content increased during storage but was reduced in the presence of EGTA-AM (**a**). Both EDTA and EGTA treatments decreased ΔΨ_M_ (**b**) and increased ATP content (**d**), whereas EGTA-AM had no effect on ATP. Oxygen consumption increased by EGTA and EGTA-AM treatments (**c**). Data are expressed as means ± SEM (n = 5, respectively). ^a–d^*P* < 0.05.

Based on previous observations of mitochondrial dysfunction during chicken semen storage^25^, we examined the effects of Ca^2+^ chelation on ROS content, ΔΨ_M_, oxygen consumption, and ATP levels at 72 h storage. ROS levels showed a significant increase in the BPSE (control), EDTA, and EGTA groups (*P* < 0.05; Fig. 3a). In contrast, ROS levels were significantly lower in the EGTA-AM group, suggesting that intracellular Ca^2+^ chelation mitigates oxidative stress. The ΔΨ_M_ was decreased in all chelator-treated groups, except for EGTA-AM, which did not show any difference from the control (Fig. 3b). Oxygen consumption was markedly increased by the mitochondrial uncoupler CCCP but not by antimycin A treatment in BPSE, as shown by oxygen-sensitive probe measurements, suggesting that liquid storage suppresses the mitochondrial electron transport chain (ETC) responsible for ATP synthesis. Notably, both EGTA and EGTA-AM increased oxygen consumption similar to the uncoupler (Fig. 3c), indicating intracellular hypoxia is induced by Ca^2+^ chelation. The ATP content was elevated in the EDTA and EGTA groups, although no significant difference was observed between the EGTA-AM and the control group (Fig. 3d). These findings demonstrate that there is differential regulation of mitochondrial activities by extracellular vs intracellular Ca^2+^ chelation.

It is widely recognized in various mammalian and avian species that sperm ΔΨ_M_, oxygen consumption, ATP content, and fertilizability are positively correlated^28,29^. However, our results contrast with this notion, as the EGTA group exhibited low ΔΨ_M_, high oxygen consumption, and high ATP levels, whereas the EGTA-AM group showed no change in ΔΨ_M_ and ATP but exhibited high oxygen consumption, with both leading to prolonged fertilizability during liquid storage.

### Ca^2+^ chelation prevents storage-induced cytoplasmic alkalosis and regulates ATP catabolism

**Figure 4:**
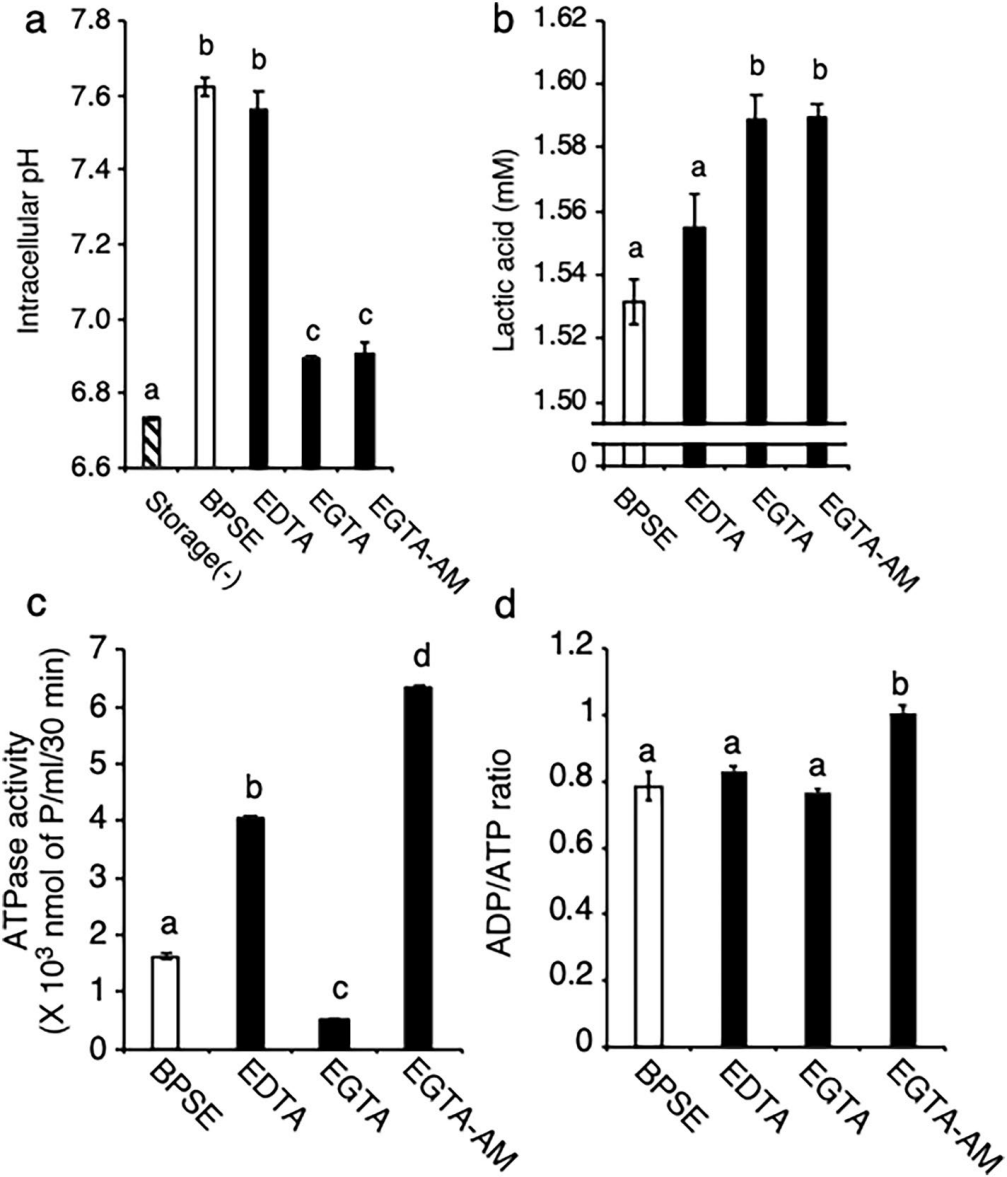
Changes in energy metabolism in chicken sperm during liquid storage. Sperm were incubated at 4 °C for 72 h with 2 mM EDTA, 2 mM EGTA, 10 µM EGTA-AM or 10 µM TPEN and then [pH]i, lactic acid content, dynein ATPase, ADP/ATP ratio were measured. Liquid preservation increased [pH]i, but this was cancelled by EGTA and EGTA-AM (**a**), concomitantly with increase in lactic acid content (**b**). ATPase activity increased upon EDTA and EGTA-AM treatments but was reduced by EGTA (**c**). EGTA-AM treatment increased ADP/ATP ratio (**d**). Data are expressed as means ± SEM (n = 5, respectively). ^a–d^*P* < 0.05.

Although our results indicate a preservation effect of poststorage fertility through mitochondrial regulation by Ca^2+^ chelation, the underlying mechanisms remain unclear. In sea urchin sperm, intracellular pH (pHi) plays a role in regulating mitochondrial respiration and dynein ATPase activity, which are responsible for cytoplasmic ATP accumulation^30^. In Japanese quail, cytoplasmic acidification through lactic acid accumulation was found to enhance sperm quiescence and prolong sperm longevity in SSTs^12^. Therefore, we examined the effects of Ca^2+^ chelation on pHi and lactic acid content in sperm at 72 h poststorage. Our measurements showed that pHi markedly increased during storage in the BPSE controls and EDTA groups compared to the nonstorage condition (7.62 and 7.56 vs. 6.73; Fig. 4a), indicating storage-induced intracellular alkalization. However, EGTA and EGTA-AM inhibited this alkalization (6.89 and 6.91, respectively). Corroborating this finding, higher accumulation of lactic acid was observed under the EGTA and EGTA-AM groups (both 1.59 mM; Fig. 4b). To examine if there was any influence of extracellular pH (pHe) on pHi, we measured pHe at 0 and 72 h storage, and found a significant reduction of pHe albeit to the same extent in both groups (with ranges 7.02–7.03 and 6.44–6.56 respectively; Supplementary Fig. S3). These results suggest that cytoplasmic alkalosis is an adverse effect of sperm storage, which can be counteracted by lactic acid accumulation induced by Ca^2+^ chelation without an impact from pHe.

Dynein ATPase is the primary consumer of ATP in sperm and is regulated by both pHi acidification and [Ca^2+^]i in humans^31^. Therefore, we hypothesized that Ca^2+^ chelation during liquid storage affects sperm dynein ATPase activity, resulting in differences in ATP content. To test this hypothesis, we analyzed sperm at 72 h storage and found that dynein ATPase activity decreased with EGTA treatment and markedly increased with EGTA-AM treatment (Fig. 4c). In these treatments, mitochondrial ATPase activity that remained the same between groups (Supplementary Fig. S4) was inhibited. Treatment with ciliobrevin that inhibits dynein ATPase negated the differences observed in ATPase activity between BPSE (control), EGTA and EGTA-AM groups confirming that the effect is specific for dynein ATPase (Supplementary Fig. S4). Analysis of ADP/ATP ratio (Fig. 4d), also revealed a relative abundance of ADP to ATP in the EGTA-AM group but not different for the BPSE (control), EDTA, and EGTA groups. Taken together with the differential effects of EGTA and EGTA-AM, these results indicate that pHi and intracellular Ca^2+^ availability regulate dynein ATP hydrolytic capacity during liquid storage. Overall, the findings of this study indicate that both extracellular and intracellular Ca^2+^ chelation can stimulate intracellular hypoxia and acidification but differentially regulate the mitochondrial ETC and dynein ATPase activity, which are primary drivers of ATP accumulation during liquid storage. Additionally, intracellular Ca^2+^ chelation appears to contribute towards the mitigation of oxidative stress. These differential mechanisms, activated in response to EGTA and EGTA-AM during liquid storage, enhances the in vitro preservation of chicken sperm fertilization potential.

## Discussion

Liquid storage of poultry semen has long presented a challenge due to gaps in understanding of physiological factors that can provide biomimicry to sustain fertility in vitro. The present study evaluates Ca^2+^ as a factor that compromises semen quality with findings that chelation of both extracellular and intracellular Ca^2+^ using EGTA and EGTA-AM respectively, potentiate the in vitro preservation of chicken semen fertility. Specifically, the chelation prolongs sperm fertilization potential through regulatory mechanisms that coordinate mitochondrial respiration and ATP catabolism. These findings not only make possible applications advancing liquid storage of poultry semen, but also provide further insights into several other previously reported observations.

In chickens, previous work has shown that sperm motility can be preserved for up to 5 h during storage by inactivating sperm using BAPTA and subsequently restoring motility through Ca^2+^ addition^25^. Consistent with preservation, the addition of Ca^2+^ elevated various motility parameters in sperm where extracellular or intracellular Ca^2+^ was previously chelated. Compared to BAPTA type chelators (*K*_d_; > 440 nM), EDTA, EGTA and EGTA-AM tested in this study possess higher binding affinity to calcium (*K*_d_; 20, 60, and 70 nM respectively)^32,33^. In the female reproductive tract, once ejaculated sperm enter SSTs, they become transiently quiescent in terms of progressive motility and mitochondrial respiration until when they emerge from the SSTs for ascent towards the egg^34,35^. Interestingly, several mammals exhibit a similar system, where ejaculated sperm can prolong viability and function by residing in sperm reservoirs along with the oviduct through interactions with epithelial cells^36–38^. Mechanistic studies on oviductal storage of boar sperm have revealed that sperm bound to the epithelium suppress motility by reducing [Ca^2+^]i^39–42^. It was previously hypothesized that reversible sperm quiescence in birds involves membrane interactions between sperm and SST epithelial cells^43^. Building on this primise, our finding regarding the prolongation of chicken sperm function in vitro through Ca^2+^ chelation provides a foundation for elucidating the mechanism underlying prolonged sperm storage in chicken SSTs, and indicate the need for further investigation on Ca^2+^ homeostasis in the resident sperm of the SSTs.

Previous studies have suggested that increased Ca^2+^ permeability during cooling storage could have deleterious effects on sperm motility and fertilization potential in poultry^21,25,44^. Our results support this notion, demonstrating that an increase in [Ca^2+^]i negatively regulates IPVL penetrability in a storage period–dependent manner. Considering that previous studies in poultry have shown that spontaneous AR is induced in response to Ca^2+^ influx^27^ and is associated with impaired poultry semen quality during in vitro storage^45^, we evaluated the effect of Ca^2+^ chelation on AR, IPVL penetrability, and in vivo fertility. Consistent with our IPVL penetration assay, our results showed that the addition of EGTA and EGTA-AM completely inhibited spontaneous AR and prolonged semen fertility, particularly observed at the 2nd week after artificial insemination. This effect was specific to Ca^2+^, as Zn^2+^ chelation using TPEN induced an adverse effect of enhanced spontaneous AR. This effect of zinc agrees with previous results that intracellular and extracellular Zn^2+^ contributes to sperm mobility and fertilizability via various pathways in poultry and mammals^46,47^. The specific effect of blocking Ca^2+^-mediated signaling events on the prologation of sperm fertilizability does not appear completely conserved uncovering nuances to signaling across genera. In stallion semen, storage conditions where intra-cellular Ca^2+^ was chelated using BAPTA-AM improved sperm viability and motility, but did not preserve fertility following by artificial insemination^26^. This is not surprising because in vivo oviductal sperm reservoirs in mammals do not provide functional quiescence to quarantined sperm, but rather potentiate capacitation-associated changes^48^. In avian species, capacitation is not required for sperm to acquire fertilizability. Therefore, the present study highlights species specificity of sperm storage that might be aligned with events within the female genital tract, and offers a novel approach to in vitro liquid preservation of poultry sperm.

Previous studies on in vitro semen storage in various species have indicated that sperm mitochondria experience failure under low temperature conditions, leading to reduced sperm fertilizability during storage^22,25,26,49^. Nevertheless, in mechanistic terms, our understanding of how sperm metabolism during cold storage relates to impaired fertility remains limited. Mitochondria are the primary source of ROS in sperm. We observed a significant increase in cytoplasmic ROS levels during liquid storage, which was partially alleviated only by EGTA-AM treatment. The main source of ROS generation is the premature leak of electrons from the ETC occurring in the inner mitochondrial membrane. Consistent with this, sperm ROS production is known to be negatively correlated with ΔΨ_M_, which represents mitochondrial ETC activity and ATP production^50^. In the present study, we observed a decrease in ΔΨ_M_ with EDTA and EGTA treatment, with a more pronounced reduction due to EGTA, despite no change being observed following EGTA-AM treatment. These results, along with the prolongation of semen fertility observed with Ca^2+^ chelation, challenge the long-standing dogma that low ΔΨ_M_ combined with high ROS levels is a primary driver of reduced sperm fertilizability in both fresh and in vitro*–*preserved sperm of mammals and birds^18,51^. Our findings with EGTA-AM suggests that the mitochondrial ETC alone cannot fully explain semen fertility poststorage and that other upstream regulatory cascades and interactions might be involved in loss of sperm fertilization potential.

Oxygen consumption is involved in the final step of the respiratory chain, where reducing equivalents from some electron donors are transported to oxygen to form water. The mitochondrial ETC and oxygen consumption are major components of mitochondrial respiration and are closely associated with ATP synthesis. The ΔΨ_M_ and oxygen consumption are known to be positively correlated with ATP content and fertilizability in mammalian sperm^52^. Interestingly, using sperm at 72 h storage, we found that Ca^2+^ chelation resulted in sperm oxygen consumption reaching levels similar to that of CCCP treatment–induced mitochondrial uncoupling in control sperm stored in BPSE. This is in agreement with previous studies with different species showing that mitochondrial O_2_ consumption is indicative of the post-storage sperm fertilizability in stallion^53^ and that luminal hypoxia of SSTs plays a role in the prolongation of quail sperm lifespan^54^. However, ATP quantification in our study revealed an increase with EDTA and EGTA treatment but not EGTA-AM treatment. Given this discrepancy, our results regarding mitochondrial and fertilizability-associated functions poststorage are not aligned with the notion of positive correlations among ΔΨ_M_, oxygen consumption, sperm ATP content, and fertilization potential in mammals^52^. Although information on poultry sperm metabolism is limited, our results in conjunction with a previous study reporting inhibition of several tricarboxylic acid cycle (TCA) enzymes through a high adenylate energy charge, representing the relative abundance of ATP to other adenine nucleosides^55^, suggests that ATP content can negatively regulate ΔΨ_M_ in poststorage chicken sperm. This view is supported by our ADP/ATP ratio assay results, which showed lower ATP abundance compared with ADP in the EGTA-AM group than in the EDTA and EGTA groups.

Sperm ATP levels are primarily controlled by mitochondrial synthesis and hydrolysis through dynein ATPase. Previous studies have shown that the inactivation of dynein ATPase via sperm acidification plays a role in sperm quiescence in the reproductive tracts of various species, including quail^54,56,57^. Our pHi analyses revealed sperm alkalization during storage in BPSE (control). Consistent with previous findings that cytoplasmic alkalization increases Ca^2+^ permeability in human sperm^58^, we observed Ca^2+^ influx during liquid storage that could be a result of cytoplasmic alkalization. Although the mechanism behind storage-induced alkalization remains unclear, this is in line with a previous notion that alkaline pH of chicken UVJ and oviduct helps maintaining high sperm mobility^59,60^. In contrast, it is notewothy that both EGTA and EGTA-AM, as Ca^2+^ chelators, inhibited this alkalization. Although semen pHe decreased in all groups to same extent at 72 h poststorage, we found the accumulation of lactic acid in response to EGTA and EGTA-AM, suggesting that the potentiation of sperm quiescence under Ca^2+^ chelation may involve lactic acid accumulation. Although increases in lactic acid appeared modest, the intracellular concentrations could be significant as sperm contain very little cytoplasm. Addition of extracellular lactic acid has been shown to have a direct influence on pHi and sperm motility in Japanese quail^54^.

Without further investigation, the differential response of ATP levels to EGTA and EGTA-AM groups cannot be fully explained. ATPase assays delineating mitochondrial vs dynein ATPase revealed the specific inhibition of dynein ATPase through EGTA-induced cytoplasmic acidification, as was expected. Surprisingly, EGTA-AM treatment led to a significant increase in dynein ATPase activity, contrary to the inhibitory effect of EGTA. This was confirmed by our ADP/ATP ratio assay, which revealed enhanced ATP hydrolysis in the EGTA-AM group. A previous study characterizing ATPase in human sperm showed that cytoplasmic acidification and Ca^2+^ reduce dynein ATPase activity^31^. Similar inhibitory effects of Ca^2+^ on dynein ATPase activity associated with microbe cilia have also been reported^61^. Taking these results together, we can conclude that elevated ATP content due to EGTA treatment primarily results from the inhibition of dynein ATPase caused by lactic acid and lower pHi. However, intracellular Ca^2+^ chelation by EGTA-AM appears to activate dynein ATPase and shows lower ATP content, but still measures elevated lactic acid and low pHi. This divergent mechanism might be indicative of an intercalated yet distinct responses linked to both glycolytic production and malleable thresholds or feedback regulation for lactic utilization via the TCA cycle that remain to be fully elucidated.

To the best of our knowledge, this study demonstrates the relevance of arresting Ca^2+^ regulated mechanisms reservation of poultry sperm fertilizability after prolonged in vitro storage through extracellular and intracellular Ca^2+^ chelation. Although the precise mechanisms are not clear, our findings indicate differential effects on mitochondrial OxPhos and ATP catabolism, both of which contribute to the preservation of poultry sperm fertilizability during in vitro storage. A previous study using Japanese quail sperm preservation through artificial insemination insinuated that preservation might not be solely dependent on cytoplasmic acidification but also involves other mechanisms^54^. Our findings advance this consideration under the umbrella of a multifaceted link between arresting Ca^2+^ signaling and avian sperm quiescence. Beyond the mechanistic considerations revealing cellular and metabolic basis of sperm functional regulation, our results contribute to the development of a semen preservation technology enables avian sperm to maintain fertilizability during in vitro storage.

## Materials and methods

### Reagents and animals

All chemicals were purchased from Sigma-Aldrich (St. Louis, MO, USA) unless otherwise noted. 2’,7’-dichlorodihydrofluorescein diacetate (DCFDA)/H2DCFDA-cellular ROS assay kit and 2’,7’-bis-(2-carboxyethyl)-5(6)-carboxyfluorescein (BCECF-AM) were from Abcam (Cambridge, UK). 5,5’,6,6’-tetrachloro-1,1’,3,3’-tetraethylbenzimidazolylcarbocyanine iodide (JC-1) was from Cayman Chemical (Ann Arbor, MI, USA). Lactate assay kit-WST was from (Dojindo Lab, Kumamoto, Japan). “Cellno” ATP assay reagent Ver.2 was from TOYO B-Net Co., Ltd (Tokyo, Japan).

The birds used in this study were fertile Rhode Island Red (RIR) YC line from the genetic stock of National Livestock Breeding Center (Aichi, Japan), and maintained in individual battery cages with 14L/10D photoperiod and ad libtum access to a commercial male diet (ZEN-NOH, Japan) and water. RIR roosters (8–19 mo. of age) were used for semen collection, which was achieved via the dorsal-abdominal massage method^62^. All procedures involving animals were performed in accordance with ARRIVE guidelines^63^, and in accordance with the livestock management guidelines of national animal welfare authorities, and were approved by the Institutional Animal Care and Use Committee (IACUC), University of Tsukuba (Approval no. 18–349).

### Semen collection, liquid storage, and treatments

Ejaculated semen from four males randomly selected from ten roosters was pooled, and the secretory fluids were separated via centrifugation at 500 *g* and room temperature for 10 min. The sperm pellet was resuspended in Beltsville Poultry Semen Extender (BPSE, pH7.3, 320 mOsm/kg) to achieve a final concentration of 2 × 10^9^ cells/ml. Liquid-state storage was performed following a previously described method^64^. Briefly, the samples (500 µL) were transferred to a microtube (509-GRD, Thermo Scientific, IL, USA) and stored under aerobic conditions with gentle shaking using a Minishaker (Kenis Ltd., Japan) at 4 °C for 0, 24, 48, 72, 92, or 120 h. During storage, the following chelators were supplemented: 2 mM ethylenediaminetetraacetic acid (EDTA), 10 µM N,N,N′,N′-tetrakis(2-pyridylmethyl)ethylenediamine (TPEN), 2 mM ethylene glycol-bis(2-aminoethylether)-N,N,N′,N′-tetraacetic acid (EGTA), or 10 µM 3,12-bis[2-[(acetyloxy)methoxy]-2-oxoethyl]-6,9-dioxa-3,12-diazatetradecanedioic acid, 1,14-bis[(acetyloxy)methyl] ester (EGTA-AM). Poststorage sperm were obtained from same samples and diluted to the appropriate sperm concentration using N-Tris(hydroxymethil) methyl-2-aminoethanesulfonic acid (TES)–NaCl buffer [20 mM TES and 150 mM NaCl (pH 7.4)] for further analysis.

### Viability and motility assessment

For viability assessment, samples (1 × 10^7^ cells/ml) were treated with 10 µM propidium iodide (P3566, Thermo Scientific) for 5 min. A minimum of 200 sperm from each sample were evaluated following the manufacturer’s instructions.

For motility assessment, samples (1 × 10^7^ cells/ml) were incubated for 10 min at 39 °C in TES–NaCl buffer, with or without 5 mM Ca^2+^. The motility profile of the samples was analyzed using a sperm motility analysis system (SMAS; version 3.19, DITECT, Tokyo, Japan), equipped with × 10 positive-phase contrast microscope (E200, Nikon, Tokyo, Japan), that records images with 1920 × 1080 pixels for 1 s in each field view. The instrumental settings were as follow; frame rate = 150 frames/s, shutter speed = 1/200, cell size = 20–150 pixels, head circularity = 0.2–2, head oblateness = 1–9, classified as motile = centroid location movement > 4.45 pixels. At least 600 sperm per sample were viewed across 4–5 field views and examined for various motility parameters, including total motility (%), straight-line velocity (VSL; μm/s), curvilinear velocity (VCL; μm/s), average path velocity (VAP; μm/s), linearity (LIN; VSL/VCL × 100), straightness (STR; VSL/VAP × 100), amplitude of lateral head displacement (ALH; μm), and beat-cross frequency (BCF; Hz).

### Intracellular calcium ([Ca^2+^]i) quantification

Samples (1 × 10^7^ cells/ml) were treated with 5 µM Fluo 4–AM (Thermo Scientific) in TES–NaCl buffer for 30 min at 37 °C, and washed by centrifugation at 2000 *g* for 3 min, and the pellets were resuspended in same buffer. In time course experiment, TES–NaCl buffer contained 1 mM Ca^2+^, and maximum and minimum fluorescence were determined using 5 µM calcium ionophore A23187 and 1 mM EDTA. Fluorescence intensity was measured using a Safire® microplate reader (Tecan, Mannedorf, Switzerland) at 485 nm excitation and 520 nm emission wavelengths.

### Inner perivitelline layer penetration assay

The sperm’s ability to penetrate the inner perivitelline layer (IPVL) was assessed following a previously described method^65^. Our ability to separate intact IPVL from outer perivitelline layer was previously confirmed by comparison of protein composition and immunoreactivity against ZPC, one of the major constituents of the avian IPVL^27^. For IPVL penetration assay, samples (1 × 10^7^ cells/ml) were coincubated with a piece of the IPVL (1 cm^2^) in TES–NaCl buffer containing 5 mM Ca^2+^ for 10 min at 39 °C. After washing with PBS, the IPVL was fixed with 4% paraformaldehyde for 15 min, gently transferred onto a glass slide, and photographed using a Leica DMI4000 B microscope (Leica Microsystems, Wetzlar, Germany). The number of holes per 0.29 mm^2^ of IPVL was calculated using ImageJ software (Ver. 1.47).

### Spontaneous AR

Samples (1 × 10^7^ cells/ml) in TES–NaCl buffer containing 5 mM Ca^2+^ were incubated for 30 min at 39 °C, after which they were treated with fluorescein isothiocyanate-conjugated peanut agglutinin for 10 min. Reacted acrosome was identified in at least 200 sperm from each sample, using a fluorescent microscope and FITC-PNA that binds to the acrosome^66^.

### Intracellular pH (pHi) and extracellular pH (pHe) measurements

Samples (1 × 10^7^ cells/ml) were treated with 5 μM BCECF-AM in TES–NaCl buffer containing 5 mM Ca^2+^ for 40 min at 39 °C. Fluorescence intensity was then measured using a DTX800 Multimode Detector (Molecular Devices, Sunnyvale, CA, USA) at excitation wavelengths of 450 nm (F1) and 500 nm (F2) and an emission wavelength of 535 nm. The intracellular pH (pH_i_) was determined using the ratio of BCECF fluorescence (F2/F1), which was calibrated using a calibration curve. To construct the calibration curve, samples were incubated in 5 μM BCECF-AM and a KCl buffer (130 mM KCl, 10 mM NaCl, 1 mM MgSO_4_, and 10 mM Na-MOPS) containing 5 μM nigericin at various pH levels (6.4, 7.0, 7.2, 7.4, and 7.8) and 39 °C for 40 min. To measure pHe, 50 μl samples with no dilution were centrifuged at 10,000 *g* for 10 min at 4 °C, and supernatants were used for pH measurement using a LAQUAtwin pH meter (HORIBA, Kyoto, Japan) installed with Sampling Sheet B (ADVANTEC, Tokyo, Japan).

### Lactic acid quantification

The quantification of intracellular lactic acid content was performed using a Lactate Assay Kit-WST (Dojindo Lab) following the manufacturer’s instructions. Samples (2 × 10^7^ cells/ml) were centrifuged at 1000 *g* for 10 min at 4 °C and resuspended in TES–NaCl buffer containing 0.1% Triton X-100. Deproteinization of the samples was performed using a centrifugal filter (Amicon Ultra 10 KDa-cutoff, Millipore, MA, USA) according to the manufacturer’s guidelines. The eluents were mixed with a working solution for the colorimetric reaction, and the absorbance was measured at a wavelength of 450 nm using a DTX800 Multimode Detector.

### Measurement of reactive oxygen species (ROS) content

Samples (1 × 10^7^ cells/ml) were incubated with 20 µM DCFDA in a assay buffer for 35 min at 37 °C in the dark. Fluorescence intensity was measured using a DTX800 Multimode Detector at excitation and emission wavelengths of 485 and 535 nm, respectively.

### Assessment of mitochondrial activity

Mitochondrial activity was assessed using JC-1, a lipophilic fluorescent probe that undergoes reversible fluorescence changes from green (monomeric form) to orange (aggregate form) with increasing mitochondrial membrane potential (ΔΨ_M_). Sperm (2 × 10^7^ cells/ml) were incubated in Ca^2+^-free TES–NaCl buffer with or without 10 µM carbonyl cyanide *m*-chlorophenyl hydrazine (CCCP) for 10 min at 39 °C. The samples were washed and then incubated with 1 μM JC-1 for 15 min at 39 °C. Fluorescence intensity of JC-1 monomers (excitation: 485 nm; emission: 530 nm) and aggregates (excitation: 535 nm; emission: 590 nm) were detected using a DTX800 Multimode Detector. The fluorescence intensity ratio (590/530 nm) was used to evaluate ΔΨ_M_.

### ATP quantification

Samples were washed and then resuspended in TES–NaCl buffer containing 5 mM Ca^2+^. Samples (6 × 10^4^ cells) were solubilized in ATP assay reagent for 10 min at 23 °C, and the luminescence signal was measured using a DTX800 Multimode Detector.

### Oxygen consumption measurement

Sperm oxygen consumption was assessed using spectrofluorometry. An Extracellular OCR Plate Assay Kit (Dojindo, Kumamoto, Japan) was used to estimate cellular oxygen consumption by measuring extracellular oxygen levels based on the phosphorescent signal of an oxygen-sensitive probe quenched under hyperoxic conditions^67^. According to the manufacturer’s instructions, a working solution was prepared by adding the oxygen probe to BPSE including no additive, 2 mM EGTA, or 10 µM EGTA-AM at a concentration of 1.1% (v/v). At 72 h poststorage with no additive, EGTA, or EGTA-AM, the samples (5 × 10^7^ cells) were centrifuged 1000 *g* at 4 °C for 5 min, after which they were resuspended in 100 µl of the respective working solutions. In positive controls, 10 µM CCCP (uncoupler) or 10 µM antimycin A [AA, electron transport chain (ETC) inhibitor] was added. The samples were added to a precooled 96-well black/clear bottom plate, covered with mineral oil, and incubated for 2 h at 4 °C. Fluorescence intensity was measured using a Safire® microplate reader at excitation and emission wavelengths of 500 nm and 650 nm. For normalization, the raw data were divided by the signal intensity obtained from the respective blank controls which contain oxygen probe ± AA or CCCP (BPSE group), + 2 mM EGTA (EGTA group) and + 10 µM EGTA-AM (EGTA-AM group) without sperm.

### ATPase activity measurement

Sperm ATPase activity was measured as described previously^54^. At 72 h poststorage, samples (5 × 10^7^ cells) were washed with assay buffer [120 mM KCl, 10 mM disodium β-glycerophosphate, 1 mM DTT, 1.8 mM MgSO_4_, 10 µM CCCP, and 10 mM HEPES (pH 7.0)]. The plasma membranes were removed by incubating the samples in the same buffer supplemented with 0.1% Triton X-100 for 2 min. After centrifugation at 10,000 *g* for 2 min, sperm pellets were resuspended in assay buffer containing 1 mM ATP and incubated for 30 min at 39 °C. For positive control, 15 µM ciliobrevin D were added to the assay buffer. The reaction was stopped by adding 34% (v/v) ice-cold trichloroacetic acid. The free phosphoric acid was allowed to react with the ferrous sulfate–ammonium molybdate reagent at room temperature for 1 min, and the absorbance was measured at a wavelength of 650 nm. A standard curve was constructed using KH_2_PO_4_ solution with various concentrations.

### ADP/ATP ratio assay

The ADP/ATP ratio was determined using an ADP/ATP Ratio Assay Kit (Sigma-Aldrich). Samples were washed and resuspended in TES–NaCl buffer, and 10 µl samples (6 × 10^4^ cells) were then solubilized with 90 µl of ATP reagent and incubated for 1 min at 23 °C. Luminescence was measured using a DTX800 Multimode Detector (RLUa). After 10 min, the reading was repeated to obtain the background signal representing the residual ATP (RLUb). ADP reagent was immediately added, and after 1 min, luminescence was measured again (RLUc). The ADP/ATP ratio was calculated using the following formula: (RLUc–RLUb)/RLUa.

### Artificial insemination

Three RIR hens (12–19 mo. of age) were used in each of the three groups, except for four hens in the 4th and 5th trials of EGTA-AM. Semen stored for 48 h with or without EGTA, and EGTA-AM was centrifuged at 1000 *g*, and the sperm pellet was resuspended in TES–NaCl containing 2 mM Ca^2+^ to obtain a final concentration of 1 × 10^9^ cells/ml. Intravaginal artificial insemination was performed by injecting 100 µl of the sample (1 × 10^8^ cells). Eggs collected from days 2–15 after insemination (199, 205, and 232 eggs in control, EGTA, and EGTA-AM groups, respectively) were incubated for 7 days in an air-forced incubator (Showa Furanki, Saitama, Japan) maintained at 37.8 °C and 70% humidity. Fertilization rates (fertile eggs/incubated eggs) were determined by candling the eggs 7 days after the start of incubation and opening clear eggs to confirm the lack in embryonic development. All hens were with minimum interval of 4 weeks between AI trials.

### Statistical analysis

Statistical analysis for multiple comparisons was performed using two-way analysis of variance (ANOVA) and one-way ANOVA followed by Tukey's honestly significant difference test. Pairwise comparisons were performed using the t-test or chi-square test with a Bonferroni correction^68^. Results were expressed as means ± standard error of the mean (SEM) and considered statistically significant with a *P*-value of < 0.05, except for the chi-square test with a Bonferroni correction, which had a significance level of *P* < 0.017.

### Supplementary Information

Supplementary Figures.

## Data Availability

The data that support the findings of this study are available from the corresponding author, AA, upon reasonable request.
