# Supplementary material for: Arresting calcium-regulated sperm metabolic dynamics enables prolonged fertility in poultry liquid semen storage
Source: Sci Rep. 2023 Dec 8;13:21775. doi: 10.1038/s41598-023-48550-2 (PMC10709635; doi:10.1038/s41598-023-48550-2)

Supplementary Figure file

Arresting calcium-regulated sperm metabolic dynamics enables prolonged fertility in poultry liquid semen storage

Pangda Sopha Sushadi[1], Maiko Kuwabara[1], Ei Ei Win Maung[1], Mohamad Shuib Mohamad Mohtar[1], Kouyo Sakamoto[1], Vimal Selvaraj[2], Atsushi Asano[3]*

[1]Graduate School of Life and Environmental Sciences, University of Tsukuba, 1-1-1 Tennodai, Tsukuba, Ibaraki 305-8572, Japan

[2]Department of Animal Science, College of Agriculture and Life Sciences, Cornell University, Ithaca, NY 14853, USA.

[3]Faculty of Life and Environmental Sciences, University of Tsukuba, 1-1-1 Tennodai, Tsukuba, Ibaraki 305-8572, Japan

*To whom correspondence should be addressed:
Atsushi Asano, PhD
Faculty of Life and Environmental Sciences, University of Tsukuba
Tennodai, Tsukuba, Ibaraki 305-8572, Japan
Email: asano.atsushi.ft@u.tsukuba.ac.jp
Tel: +81-29-853-6691

Supplementary Fig. S1

Chicken sperm motility during at 24 h poststorage. EGTA and EGTA-AM preserved some motility parameters (‡*P < 0.05* vs BPSE alone). Ca^{2+} addition enhanced motility parameters in BPSE, EDTA and EGTA-AM (**P* < 0.05 in treatments). Data are expressed as means ± SEM (n = 7, respectively).

Supplementary Fig. S2

Chicken sperm motility during at 48 h poststorage. EGTA and EGTA-AM preserved some of motility parameters (‡*P < 0.05* vs BPSE alone). Ca^{2+} addition enhanced motility parameters in BPSE, EDTA and EGTA-AM (**P* < 0.05 in treatments). Data are expressed as means ± SEM (n = 7, respectively).

Supplementary Fig. S3

pHe measurements at 0 and 72 h poststorage. At the time of addition to sperm, pHe of all medium variants measured 7.3, same as it is in the original formulation at preparation. pHe decreased to identical extents in all medium variants during 72 h storage (**P* < 0.05 between 0 and 72 h storage; n = 7).

Supplementary Fig. S4

Delineated ATPase activity under mitochondrial and dynein ATPase inhibitors. Reduced and elevated ATPase was observed in EGTA and EGTA-AM under 20 µM CCCP (†*P* < 0.05 vs BPSE alone). Including 15 µM ciliobrevin in addition to CCCP decreased ATPase activity to an identical baseline level across all treatment groups [n = 8 for treatments and n=3 for controls].

Supplementary fig 1

24 h poststorage

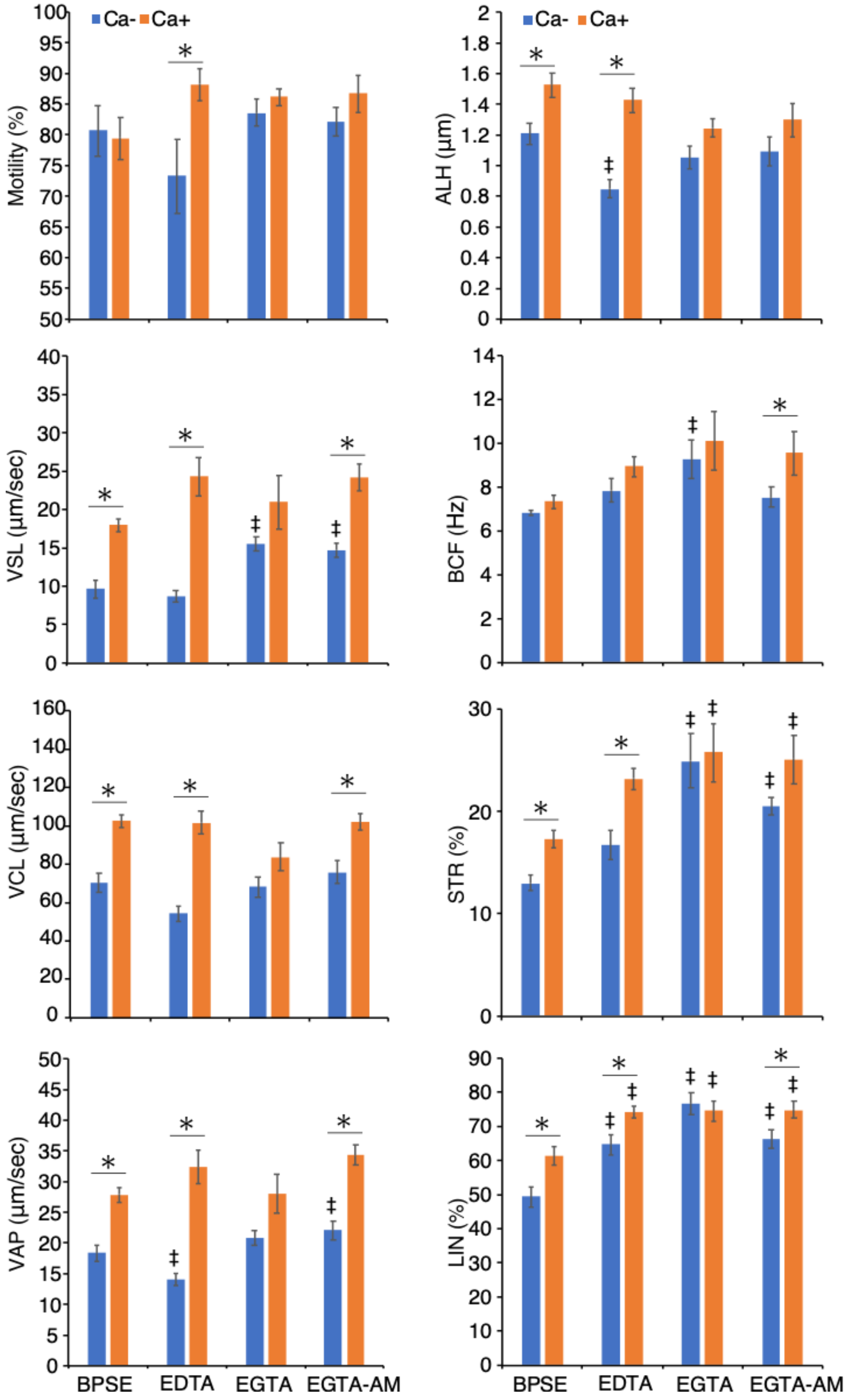

Supplementary fig 2

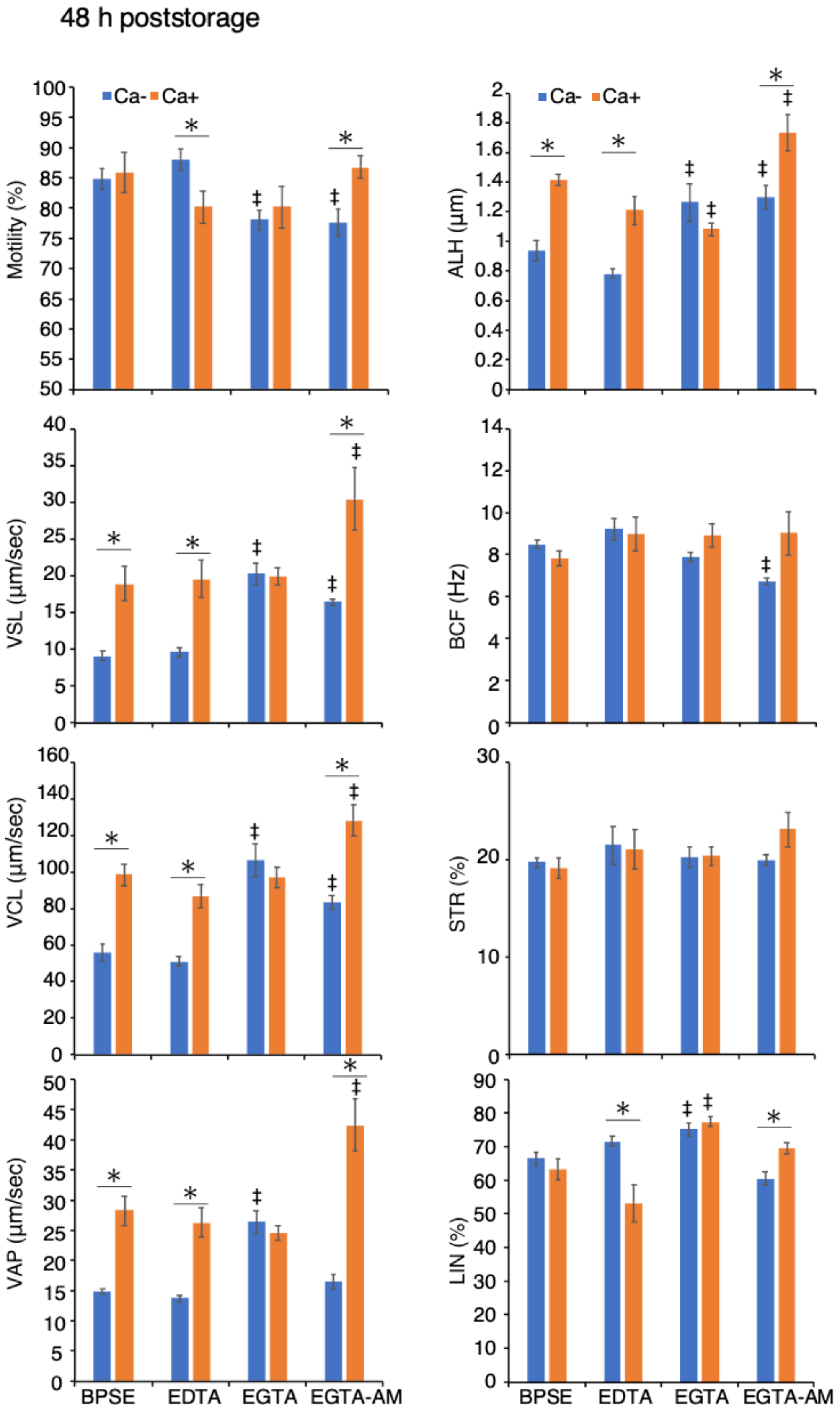

Supplementary fig 3

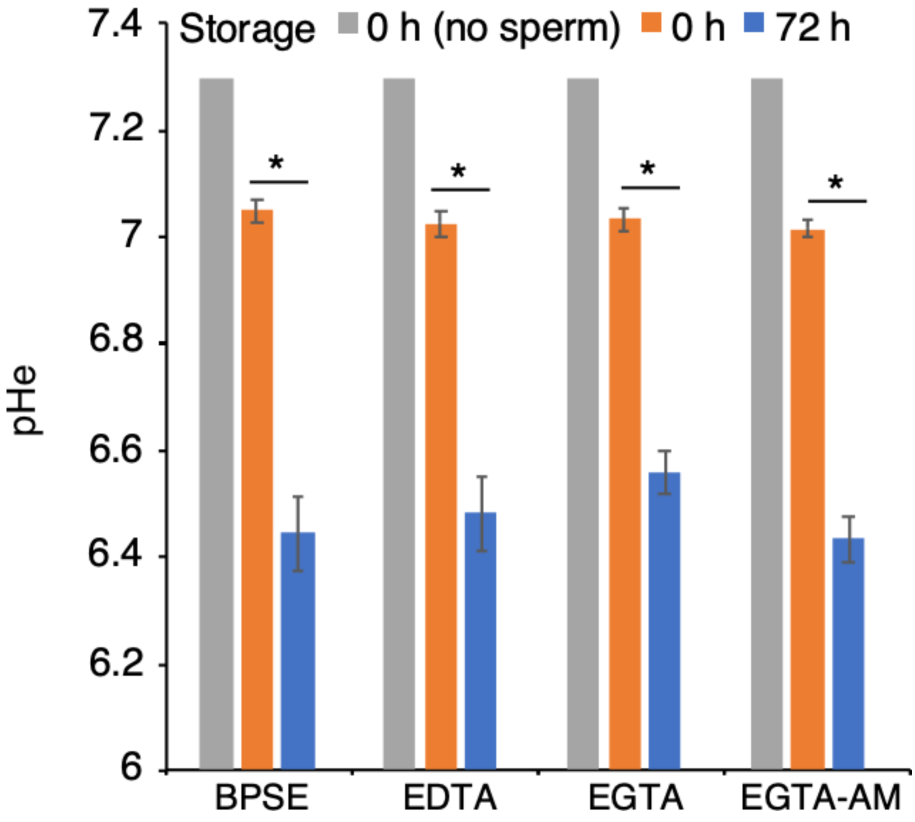

Supplementary fig 4

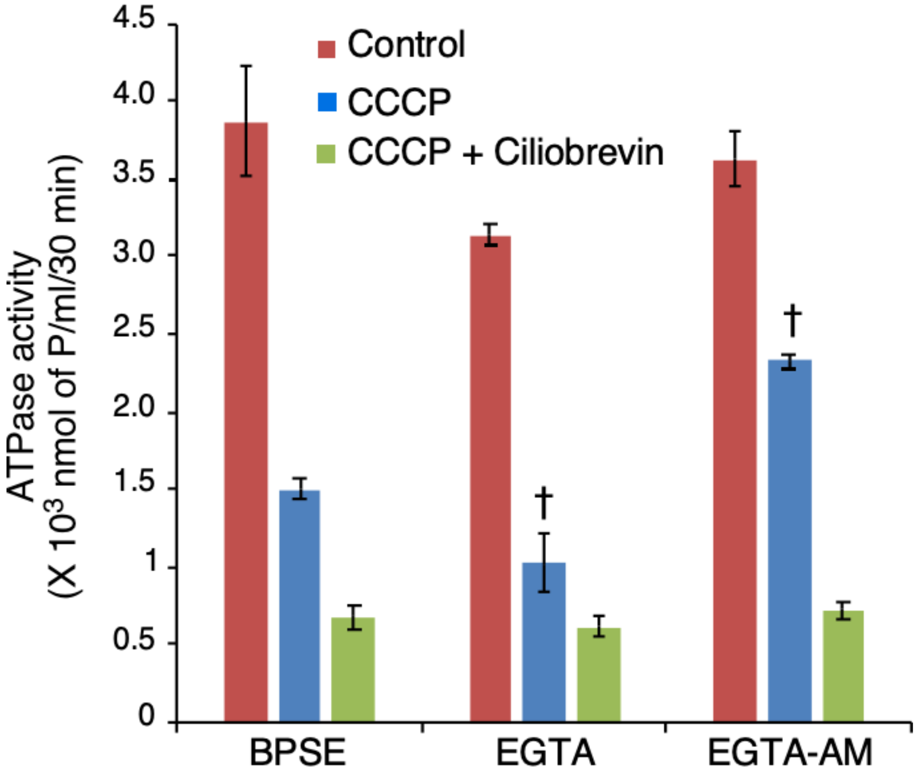

Supplement: Supplementary file 1 — Supplementary Figures. [file 41598_2023_48550_MOESM1_ESM.pdf]
